# Dual Influence of Size and Electric Field on Gold Nanoparticles: Insights into 2D Monolayer Assembly

**DOI:** 10.1021/acs.langmuir.4c03580

**Published:** 2025-01-07

**Authors:** Tholkappiyan Ramachandran, Ashraf Ali, Firdous Ahmad Deader, Hibah Shafeekali, Lianxi Zheng, Haider Butt, Moh’d Rezeq

**Affiliations:** † Department of Physics, Khalifa University of Science and Technology, Abu Dhabi 127788, United Arab Emirates; ‡ Department of Mechanical and Nuclear Engineering, Khalifa University of Science and Technology, Abu Dhabi 127788, United Arab Emirates; § System on Chip Lab, Khalifa University of Science and Technology, Abu Dhabi 127788, United Arab Emirates

## Abstract

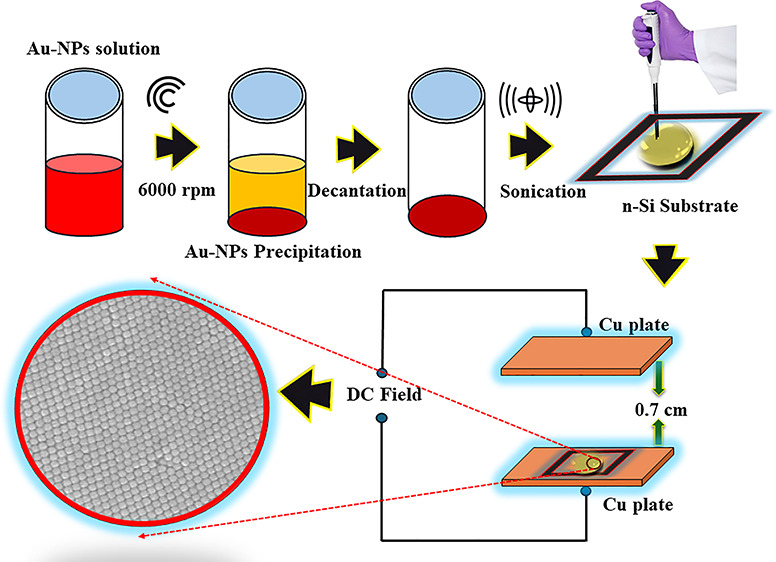

Self-assembled gold nanoparticles (Au-NPs) possess distinctive properties that are highly desirable in diverse nanotechnological applications. This study meticulously explores the size-dependent behavior of Au-NPs under an electric field, specifically focusing on sizes ranging from 5 to 40 nm, and their subsequent assembly into 2D monolayers on an n-type silicon substrate. The primary objective is to refine the assembly process and augment the functional characteristics of the resultant nanostructures. Utilizing a multifaceted analytical approach encompassing X-ray diffraction (XRD) and scanning electron microscopy (SEM) with energy-dispersive X-ray spectroscopy (EDXS), atomic force microscopy (AFM), and COMSOL multiphysics simulation, this work yields comprehensive insights. Results reveal that the electric field and nanoparticle size critically influence assembly dynamics due to variations in surface energy and electrostatic interactions. Larger Au-NPs (20, 30, and 40 nm) experience enhanced dipolar interactions and more substantial polarizability, enabling more efficient alignment and organization under an applied electric field. This leads to the formation of structured, uniform monolayers with minimal vacancies and smoother surfaces. In contrast, smaller Au-NPs (5, 10, and 15 nm) exhibit lower polarizability, which hampers alignment and promotes clustering and voids. XRD analysis delineates notable disparities in peak intensities and positions: smaller Au-NPs exhibit diminished (111) peak intensities, indicative of uneven distribution and crystallinity, whereas larger particles manifest higher intensities and well-defined peaks across multiple crystallographic planes. SEM images portray diverse surface coverages with AFM corroborating that larger Au-NPs achieve uniform and continuous monolayers with minimal height variations. COMSOL simulations substantiate these findings by illustrating the efficient alignment and settling of larger Au-NPs under the electric field. This study bridges critical gaps in understanding how nanoparticle size modulates assembly dynamics and the resultant properties of 2D Au-NP monolayers, offering pivotal insights into engineering advanced nanostructured materials tailored to specific applications in electronics, coatings, photonics, and catalysis.

## 1 Introduction

Gold nanoparticles (Au-NPs) have emerged as a cornerstone in nanotechnology due to their exceptional properties and versatile applications.^1−4^ These nanoparticles exhibit unique optical properties, such as localized surface plasmon resonance (LSPR), which results in strong absorption and scattering of light at specific wavelengths, making them ideal for applications in imaging, sensing, and photothermal therapy.^5^ Their high surface area-to-volume ratio enhances their reactivity and ability to serve as catalysts in chemical reactions.^6^ The physical chemistry of Au-NPs involves a detailed understanding of their size, shape, surface charge, and the surrounding dielectric environment, all of which critically influence their optical and electronic properties.^7,8^ Functionalization of Au-NPs with various ligands allows for tailored interactions and enhanced stability, broadening their application spectrum.^9^ Applications of Au-NPs are diverse, ranging from drug delivery systems where they facilitate targeted therapy and reduced side effects, to diagnostic tools in medical imaging due to their strong contrast properties.^10^ In catalysis, Au-NPs serve as efficient catalysts for various reactions, including oxidation and reduction processes.^11^ Their use in electronic devices, such as sensors and conductive materials, leverages their conductive properties and ability to form structured networks.

A particularly interesting aspect of Au-NP research is their self-assembly into well-defined structures. Self-assembled gold nanoparticles capitalize on noncovalent interactions like van der Waals forces, hydrogen bonding, and electrostatic interactions to form ordered arrays.^12^ The chemistry behind this self-assembly often involves surface modification with ligands that induce specific interactions, promoting controlled and predictable assembly.^13^ This process is crucial for applications requiring precise nanoscale architectures, such as in the fabrication of highly sensitive sensors, enhancement of catalytic processes, and the development of photonic devices.^14,15^ However, one of the significant challenges in the field is achieving uniform self-assembly.^16,17^ Issues such as uncontrolled aggregation and inconsistent particle spacing can impede the performance and reproducibility of self-assembled Au-NP-based applications.^18−23^ Various techniques have been developed to address these challenges, including solvent evaporation, template-assisted assembly, and the application of external fields like electric or magnetic fields.^24−28^ Among these, electric-field-assisted assembly has shown particular promise.^29^ By applying an electric field, researchers can influence the movement, orientation, and spacing of Au-NPs, thus achieving a more uniform and controlled assembly.^8,30^ This method has been reported to induce the formation of highly ordered monolayers and other structured arrays, which are essential for applications in electronics, photonics, and sensing technologies.^31,32^ The effect of nanoparticle size on the assembly process is a critical parameter that influences the properties and performance of the resultant nanostructures.^33^

Fine-tuning the size of Au-NPs has led to notable improvements in uniformity and performance, making it a vital area of research for advancing nanotechnology applications. For instance, studies by Yadavali et al.^34^ and Heyou Zhang et al.^35^ have demonstrated that larger Au-NPs tend to form more compact and uniform monolayers under an electric field, whereas smaller nanoparticles exhibit enhanced plasmonic properties but face challenges in achieving uniform spacing. The combined effects of NP size and electric field on assembly stem from size-dependent polarizability and surface energy variations that influence each NP’s response to the field. Larger Au-NPs, with increased polarizability, experience stronger dipolar interactions and align more effectively under an electric field, leading to structured, uniform monolayers with fewer voids and improved surface coverage. These particles experience less movement and adhere closely to the electric field lines, facilitating compact assembly due to their substantial interaction with the substrate and neighboring particles. Additionally, larger NPs tend to overcome thermal motion more effectively, reducing randomization in assembly and enhancing order within the monolayer structure. Conversely, smaller Au-NPs exhibit lower polarizability, which weakens their response to the electric field and disrupts the uniformity. This reduced polarizability hampers their alignment, leading to aggregation rather than ordered monolayer formation. Smaller NPs are also more susceptible to random motion and less influenced by the electric field, which, along with their higher surface energy, results in clustering and uneven surface coverage. These disparities highlight how size-dependent properties, including polarizability and dipole–dipole interactions, control the alignment, stability, and uniformity of the Au-NP assembly under an electric field. This nuanced understanding of the interplay between NP size and electric field effects advances our knowledge of the design of Au-NP assemblies, particularly for applications where precise spatial arrangement, electronic uniformity, and enhanced optical or catalytic properties are critical.

The aim of this study is to delve deeper into the size-dependent behavior of Au-NPs under an electric field, focusing on 5–40 nm nanoparticles. By investigating how these specific sizes influence the 2D monolayer assembly, we aim to optimize the assembly process and enhance the functional properties of the resulting nanostructures. Larger particles, defined as 20–40 nm Au-NPs, are characterized by stronger dipole interactions, which facilitate their alignment and lead to the formation of uniform monolayers. In contrast, smaller particles, defined as 5–15 nm Au-NPs, exhibit a tendency to cluster due to weaker interactions and higher surface energy. Through a multidimensional analysis approach utilizing X-ray diffraction (XRD), scanning electron microscopy (SEM) with energy-dispersive X-ray spectroscopy (EDXS), atomic force microscopy (AFM), and COMSOL Multiphysics simulation, the research reveals significant insights. XRD analysis highlighted distinct differences in peak intensities and positions: 5, 10, and 15 nm Au-NPs exhibited lower intensities in (111) plane peaks, indicating less uniform distribution and crystallinity, while 20, 30, and 40 nm Au-NPs showed higher intensity and well-defined peaks across multiple crystallographic planes. SEM images at 100 nm scale depicted varying surface coverage and aggregation tendencies, with smaller Au-NPs (5, 10, and 15 nm) showing noticeable surface vacancies and clustering, contrasting with larger nanoparticles (20, 30, and 40 nm) displaying minimal voids and a smoother surface. AFM analysis further confirmed these findings, illustrating that larger Au-NPs achieved more uniform and continuous monolayer formations with minimal height variations compared with smaller counterparts. COMSOL simulations supported these experimental observations, demonstrating more efficient alignment and settling of larger Au-NPs under the electric field, leading to structured monolayers. This study aims to bridge the gap in understanding how size variations influence the assembly process and the resulting properties of the 2D Au-NP monolayers. By focusing on 5–40 nm Au-NPs, we seek to provide insights that will aid in the design and fabrication of advanced nanostructured materials with tailored properties for specific applications in electronics, coating, photonics, and catalysis.

## 2 Experiemental Section

### 2.1 Materials

Citrate-stabilized Au-NPs with nominal diameters of 5, 10, 15, 20, 30, and 40 nm were acquired from Ted Pella, Inc. According to the Certificate of Analysis provided by the manufacturer, the size distributions for these particles are as follows: 5 nm (4.8 ± 0.5), 10 nm (10.2 ± 0.6), 15 nm (15.1 ± 0.7), 20 nm (20.3 ± 0.8), 30 nm (28.0 ± 0.9), and 40 nm (39.5 ± 1.0 nm). These values represent the mean particle diameter ± standard deviation, reflecting the high-quality control and consistency in the synthesis process. The n-Si-Silicon substrates, featuring a resistivity of 5–10 Ω·cm with a roughness of about 0.5 nm, were obtained from University Wafer, Inc. These substrates were cut into 1 cm × 1 cm pieces for use in subsequent experiments.

### 2.2 Preparation of Au-NPs Deposition on the Silicon Substrate

The 1 cm × 1 cm silicon substrate underwent a sequential cleaning process using acetone (≥99.8%, Sigma-Aldrich, USA), followed by ethanol (≥99.9%, Sigma-Aldrich, USA) and isopropyl alcohol (99%, Qualikems, India), rinsed with deionized water (resistivity = ≥18 × 10^6^ Ω·cm, medichem L.L.C, UAE), and ultrasonicated for 5 min in each solution. It was then dried using nitrogen gas. The drop-casting procedure was carried out with precision to achieve a monolayer of Au-NPs on the silicon substrate. After centrifuging at 6000 rpm (Eppendorf-Centrifuge 5810 R-Fisher Scientific), the Au-NP solution to increase particle concentration, the precipitated solution was subjected to 30 min of sonication to ensure a homogeneous distribution of particles. Once the Au-NPs were uniformly dispersed, a 2 μL aliquot of the concentrated solution was carefully deposited onto the center of each cleaned 1 cm × 1 cm silicon substrate using a calibrated micropipette. The substrates were placed in a high electric field setup immediately after drop-casting, allowing the electric field to influence the distribution and alignment of Au-NPs as the solution dried, facilitating uniform monolayer formation. To explore the effects of varying electric fields, different DC voltages were applied across a 0.7 cm gap between plates. The electric field (*E*) between two parallel plates can be calculated using the formula,
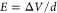
1where *E* is the electric field strength in volts per meter (V/m), Δ*V* is the applied voltage between the parallel plates, in volts (V), and *d* is the distance between the plates in meters (m). A voltage of 250 V created an electric field of ∼3.57 × 10^4^ V/m, while 500 V generated ∼7.14 × 10^4^ V/m. Increasing to 750 and 1000 V yielded electric fields of ∼1.07 × 10^5^ and ∼1.43 × 10^5^ V/m, respectively. These variations in the electric field strength allowed for controlled manipulation of Au-NP alignment and spacing, helping to achieve a consistent particle arrangement across the substrate surface.

The complete process of sample preparation, along with the experimental setup for a parallel plate capacitor structure to apply a uniform electric field on the Au-NPs solution droplet on the silicon substrate, is depicted in Figure 2. The parallel plate capacitor consists of copper plates separated by 0.7 cm. The silicon substrate is positioned at the center of the positively biased plate, and a DC voltage is applied between the plates. Figure 3 shows a schematic representation illustrating the balance of horizontal forces, which includes surface tension and electric repulsion forces. The net electric force, acting downward, is depicted as the resulting force on the system. This suggests that while the horizontal forces cancel each other out, leading to a state of equilibrium in the horizontal direction, the downward electric force remains unbalanced, resulting in a net force acting in the downward direction. The solution is allowed to dry under the electric field for approximately 45 min, after which the voltage is turned off, and the silicon substrate is removed for further characterization. The as-prepared samples named as 15, 20, 30, and 40 nm Au-NPs. We applied different voltage strategies from 250 V to 1 kV in order to form a monolayer of the prepared samples. To ensure reproducibility and consistency, around five independent substrates were prepared under each condition for each Au-NP size (15, 20, 30, and 40 nm). This approach allowed for statistical validation of the experimental results and ensured consistent monolayer formation across all tested conditions.

**Figure 1:**
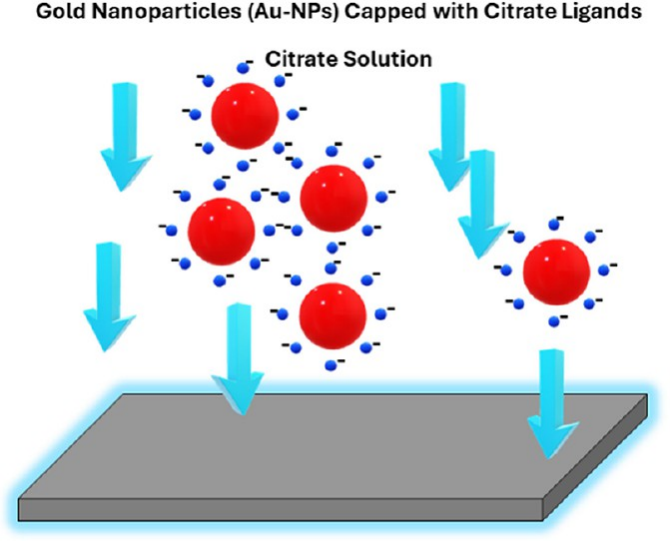
Schematic diagram illustrates the stabilization and surface functionalization of Au-NPs with citrate ligands.

**Figure 2:**
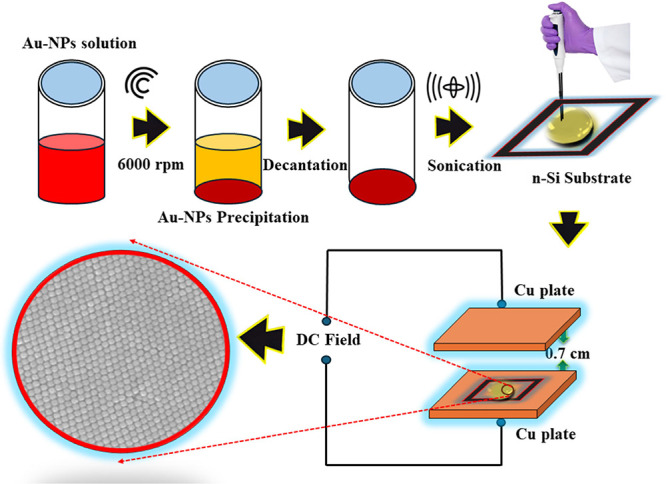
Sample preparation procedure and setup for applying an electric field to the Au-NP solution droplet on the silicon substrate.

**Figure 3:**
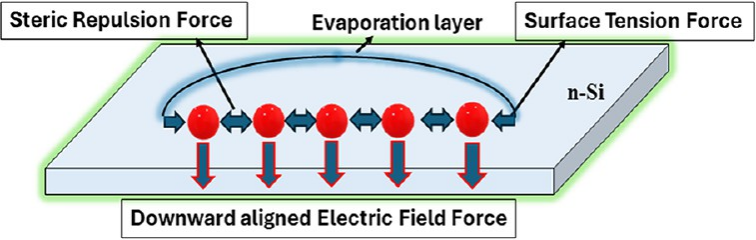
Schematic illustrating the balance of horizontal forces due to surface tension and electric repulsion with the net electric force acting downward.

### 2.3 Characterization

The Au-NP deposition on the silicon substrate underwent thorough characterization at room temperature using various techniques. Structural properties were determined through XRD analysis using a Bruker D2 phaser instrument, a desktop model from The Netherlands. The analysis utilized a Cu Kα wavelength X-ray source, operating at 30 kV voltage and 10 mA current, with a scanning range of diffraction angles from 10 to 80°. Phase identification was achieved by comparing the obtained diffraction patterns with standard data available in the ICDD (International Centre for Diffraction Data) database, ensuring an accurate assessment of the material’s crystalline structure. A JEOL JSM-7610F Schottky Field Emission Scanning Electron Microscope (FESEM) was employed to examine the microstructural features. The sample was investigated under a high level of vacuum (ULVAC KIKO Inc., The Model: G-100DB, Miyazaki in Japan), and microscopy images were captured with miXcroscopy software. The chemical composition and visualization of substances have been investigated through a JEOL-FESEM instrument furnished with an energy-dispersive X-ray detection system (EDXS). The surface morphology was examined using an Asylum Research Atomic Force Microscope (AFM). The AFM images were captured using a silicon tip AFM probe (Tap150-G cantilever) purchased from Ted Pella, Inc., optimized for tapping mode. This cantilever operated at a resonance frequency of 150 kHz with a spring constant of 5 N/m, ensuring high sensitivity and precision for the surface morphology analysis of the Au-NP assemblies. The finite element simulation model was analyzed using COMSOL Multiphysics software. The zeta potentials in an aqueous solution were determined using a Malvern zeta sizer (Malvern Instruments Ltd.).

## 3 Results and Discussion

Figure 1 shows the schematic diagram illustrating the stabilization and surface functionalization of Au-NPs with citrate ligands.

Each Au-NP, represented as a red sphere, is surrounded by citrate ligands shown as small blue dots. These citrate ligands impart a negative ζ-potential to the Au-NPs, indicated by negative (−) signs around the particles. This negative potential prevents the Au-NPs from agglomerating by causing a repulsion between them. The Au-NPs are distributed within a citrate solution for stabilization, depicted by a container labeled “Citrate Solution”. At the bottom of the schematic, a gray platform represents a silicon substrate covered with a thin native silicon oxide layer. Arrows are used to indicate the movement of Au-NPs toward this substrate under a high electric field, as they are driven by their negative ζ-potential (hence, negative charge density). The ζ-potential values in Table 1, measured using the Malvern zeta sizer, indicate that as the size of Au-NPs increases from 15 to 40 nm, the ζ-potential becomes less negative. This trend correlates with a decrease in the electrostatic repulsive forces. Smaller particles, such as 15 nm Au-NPs with a ζ-potential of −280 mV, experience significant electrostatic repulsion due to the higher charge density, leading to enhanced stability in the citrate solution. As the particle size increases, this repulsion decreases, as seen with the 40 nm Au-NPs, which exhibit a ζ-potential of −220 mV. The enhanced electric field on the NP, i.e. surface charge density, is inversely proportional to the particle size, meaning smaller nanoparticles require less external field to achieve proper alignment and deposition on the silicon substrate. Larger particles, with a lower ζ-potential, need a stronger electric field to ensure uniform monolayer formation on the silicon substrate. The differences in the ζ potential underscore how particle size directly influences both the stability of Au-NPs in solution and the external field needed for controlled assembly on the substrate.

**Table 1:** Zeta Potential (ζ-Potential) of Au-NPs at Different Sizes

Au-NP size (nm)	ζ-potential (mV)	comments
15	–280	small particle size results in higher ζ-potential, leading to stronger repulsive forces and enhanced stabilization in the citrate solution.
20	–260	a slight decrease in ζ-potential indicates reduced repulsion, but particles remain well-dispersed due to sufficient charge.
30	–230	as the particle size increases, ζ-potential drops further, requiring greater electric field strength to achieve uniform monolayer deposition.
40	–220	the lowest ζ-potential among the sizes, requiring a stronger external electric field for proper deposition and stabilization.

### 3.1 Analysis of Au-NPs Deposition on the Silicon Substrate

The influence of morphological properties on the deposition of Au-NPs of varying sizes on n-Si substrates under different applied electric fields was thoroughly investigated by using scanning electron microscopy (SEM). This analysis provided insights into how electric field strength impacts the uniformity and quality of the Au-NP deposition.

#### 3.1.1 Deposition of 5 and 10 nm Au-NPs

**Figure 4:**
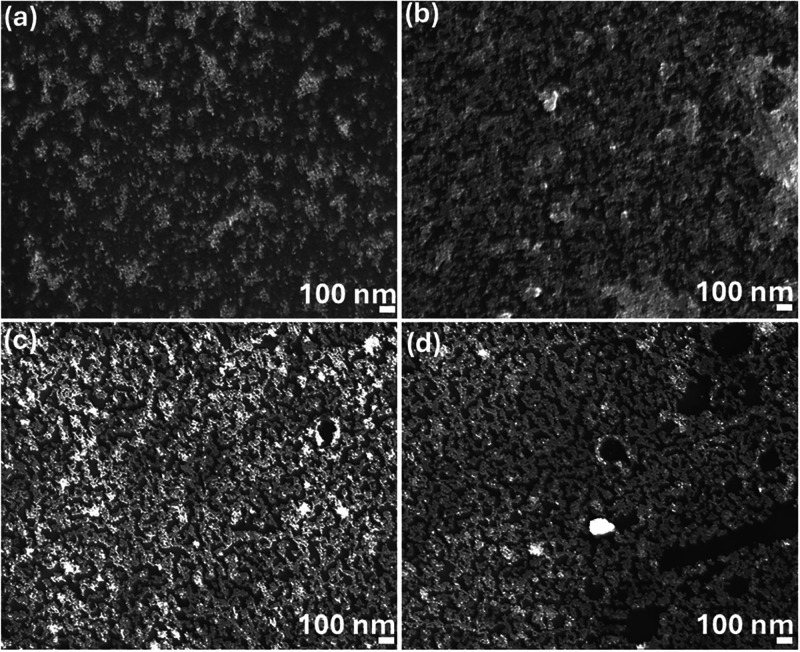
SEM images for 5 nm at (a) 250 V and (b) 500 V and 10 nm at (c) 250 V and (d) 500 V sized Au-NP deposition on the n-Si substrate.

Figure 4a–d presents the SEM images of 5 and 10 nm Au-NPs deposited on an n-Si substrate at applied voltages of 250 and 500 V. For the 5 nm Au-NPs at 250 V, as shown in Figure 4a, the deposition reveals significant gaps between particles, leading to a high degree of nonuniformity across the sample. However, when the voltage is increased to 500 V, as seen in Figure 4b, overagglomeration of the nanoparticles occurs, resulting in compromised uniformity and aggregation. Similarly, for the 10 nm Au-NPs at 250 V, as shown in Figure 4c, the deposition is nonuniform, with noticeable vacancies and voids scattered throughout the surface. Upon increasing the voltage to 500 V, as depicted in Figure 4d, the voids become more pronounced, suggesting a further decline in the deposition quality. This highlights that both voltage and nanoparticle size play pivotal roles in achieving uniform coverage.

#### 3.1.2 Deposition of 15 nm Au-NPs

**Figure 5:**
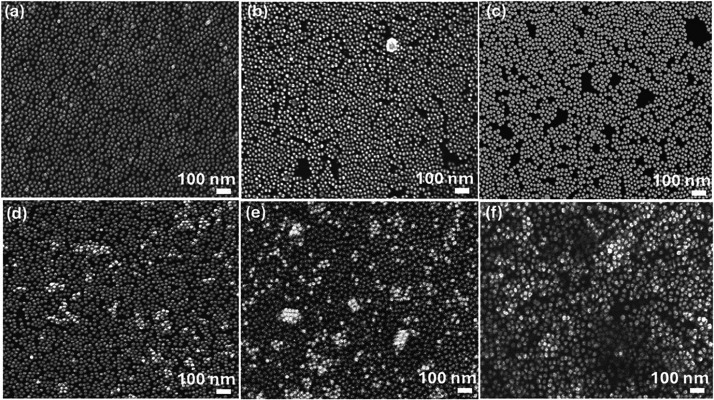
SEM images for 15 nm sized Au-NPs deposition on the n-Si substrate with different applied electric voltages: (a) 250 V, (b) 300, (c) 500, (d) 750, (e) 1000, and (f) 1.5 kV.

Figure 5a–f displays SEM images of 15 nm-sized Au-NPs deposited on the n-Si substrate with applied electric voltages ranging from 250 V to 1.5 kV. At 250 V (Figure 5a), the deposition exhibits a higher degree of uniformity, with only minor vacancies and voids. However, as the electric voltages increase to 300 V, 500 V, 750 V, 1000 kV, and 1.5 kV (Figure 5b–f, respectively), these voids become more prominent, indicating a gradual decline in deposition quality. The increasing field strength disrupts the uniform arrangement, leading to an uneven layer of Au-NPs. This suggests that while a lower field of 250 V may facilitate better monolayer formation, higher fields contribute to the nonuniformity, likely due to overagglomeration or improper deposition dynamics.

#### 3.1.3 Deposition of 20 nm Au-NPs

**Figure 6:**
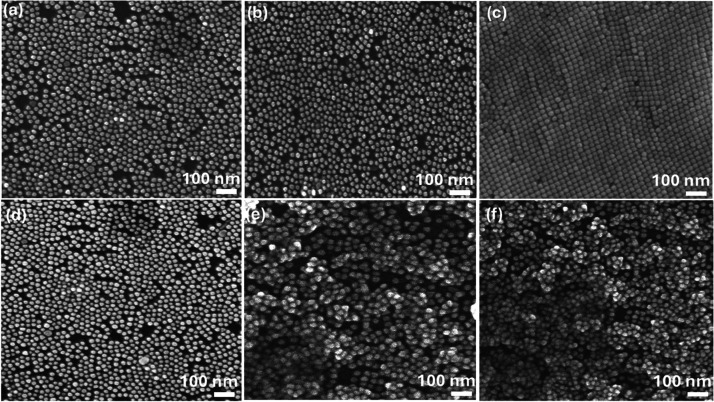
SEM images for 20 nm sized Au-NPs deposition on the n-Si substrate with different applied electric voltages: (a) 250 V, (b) 300, (c) 500, (d) 750, (e) 1000 kV, and (f) 1.5 kV.

Figure 6a–f illustrates SEM images of 20 nm-sized Au-NPs deposited on the n-Si substrate under electric voltages from 250 V to 1.5 kV. The deposition at 250 V and above, except at 500 V, reveals significant vacancies and voids, leading to nonuniform layers. Interestingly, at 500 V (Figure 6c), the deposition appears to achieve better uniformity with fewer voids, suggesting that this specific electric field is optimal for 20 nm Au-NPs. Figure 6d–f, at voltages 750, 1000, and 1.5 kV, respectively, exhibited a greater number of voids and agglomeration of NPs. This finding underscores the importance of tuning the electric field strength to achieve desirable monolayer formation, where a 500 V voltage might be more effective in preventing voids and ensuring even deposition.

#### 3.1.4 Deposition of 30 nm Au-NPs

**Figure 7:**
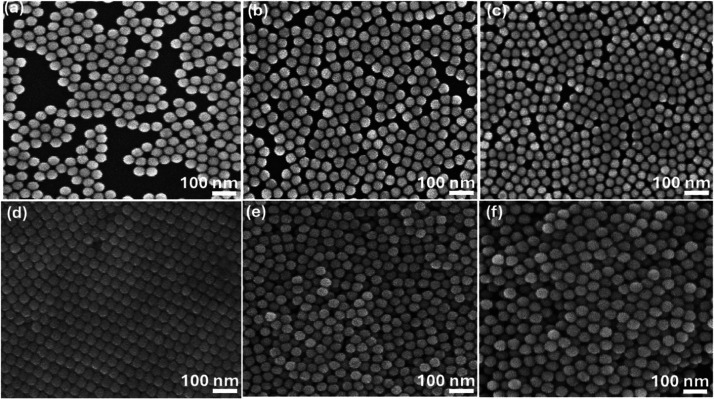
SEM images for 30 nm sized Au-NPs deposition on the n-Si substrate with different applied electric voltages: (a) 250 V, (b) 300, (c) 500, (d) 750, (e) 1000 kV, and (f) 1.5 kV.

SEM images presented in Figure 7a–d showcase the deposition of 30 nm Au-NPs under various electric fields. The sample exposed to a 250 V field (Figure 7a) shows significant surface voids and irregularities, indicative of poor deposition. As the voltages are increased to 300 and 500 V (Figure 7b,c), these voids decrease, and deposition uniformity improves. A 750 V field (Figure 7d) results in a well-organized monolayer, demonstrating an optimal deposition at this voltage. However, when the voltages are further increased to 1000 and 1.5 kV, the deposition becomes less controlled, resulting in overagglomeration and disrupted monolayer formation. This highlights the delicate balance required when applying electric fields to achieve consistent monolayer structures, particularly for larger Au-NPs like the 30 nm variety.

#### 3.1.5 Deposition of 40 nm Au-NPs

**Figure 8:**
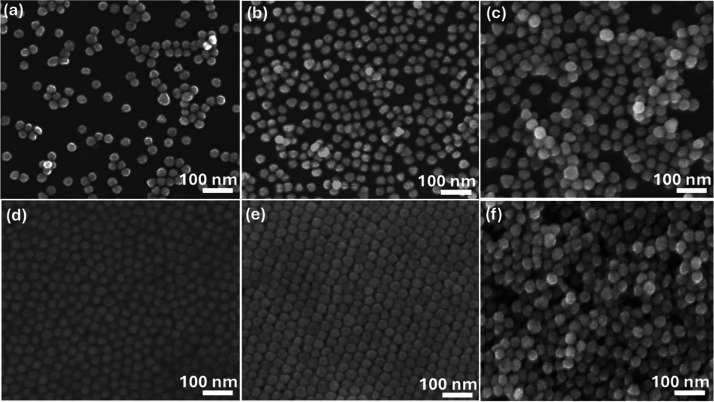
SEM images for 40 nm sized Au-NPs deposition on the n-Si substrate with different applied electric voltages: (a) 250 V, (b) 300, (c) 500, (d) 750, (e) 1000 kV, and (f) 1.5 kV.

Figure 8a–d depicts SEM images of 40 nm-sized Au-NPs deposited under electric voltages ranging from 250 V to 1.5 kV. The sample subjected to a 250 V field (Figure 8a) shows significant spaces between particles, indicating uneven deposition. Increasing the voltages to 300 V (Figure 8b) leads to close-to-one interactions and a more organized arrangement of Au-NPs, although some agglomeration begins to appear. At 500 V (Figure 8c), further agglomeration is evident but the deposition quality improves overall, suggesting the field strength is nearing optimal. Advancing to a 750 V electric field in Figure 8d, the SEM image reveals a well-structured monolayer of Au-NPs, with only minimal voids, indicating a superior deposition process. However, when the applied voltage is further elevated to 1000 kV, as seen in Figure 8e, the deposition process becomes more controlled, leading to the formation of a well-arranged Au NPs monolayer. However, as the voltage increases to 1.5 kV, overagglomeration occurs as seen in Figure 8f, leading to the formation of multiple layers and compromised uniformity.

**Figure 9:**
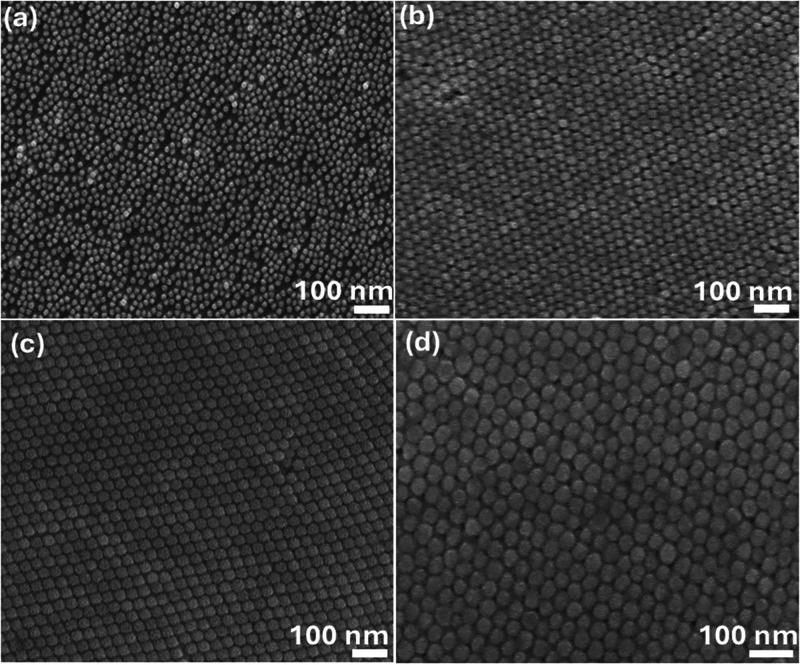
SEM images for (a) 15 nm at 250 V, (b) 20 nm at 500 V, (c) 30 nm at 750 V and (d) 40 nm at 1000 kV sized Au-NPs deposition on the n-Si substrate.

The study demonstrates that the deposition of Au-NPs on n-Si substrates is highly sensitive to both the size of the nanoparticles and the applied electric field strength. For smaller Au-NPs (15 and 20 nm), lower (250 V) to midrange (500 V) fields tend to produce better monolayers as shown in Figure 9a–d, whereas higher fields disrupt uniformity. For larger nanoparticles (30 and 40 nm), moderate fields around 750 V and 1000 kV achieve optimal deposition, but exceeding this threshold leads to overagglomeration and multilayer formation. Table 2 shows the electric field that resulted in the formation of the best monolayer for each nanoparticle size. This work underscores the importance of carefully optimizing electric field parameters to achieve desired deposition outcomes with particular attention to nanoparticle size and the corresponding electric field that yields the most uniform monolayer structure.

**Table 2:** Summarizes the Results, Highlighting the Optimal Electric Field for Achieving a Perfect Monolayer for Each Au-NP Size

Au-NPs size (nm)	voltage (V)	electric field (V/m)	deposition quality	comments
15	250	3.57 × 10^4^	optimal monolayer formation	minimal voids; other electric fields led to larger voids and nonuniform layers.
20	500	7.14 × 10^4^	high uniformity	small vacancies and voids observed, but overall a higher quality of monolayer formation.
30	750	1.07 × 10^5^	well-organized monolayer	optimal at 750 V; increasing the field further led to over-agglomeration and nonuniformity.
40	1000	1.43 × 10^5^	highly structured monolayer	best monolayer formation at 1 kV; higher fields led to agglomeration and compromised uniformity.

**Figure 10:**
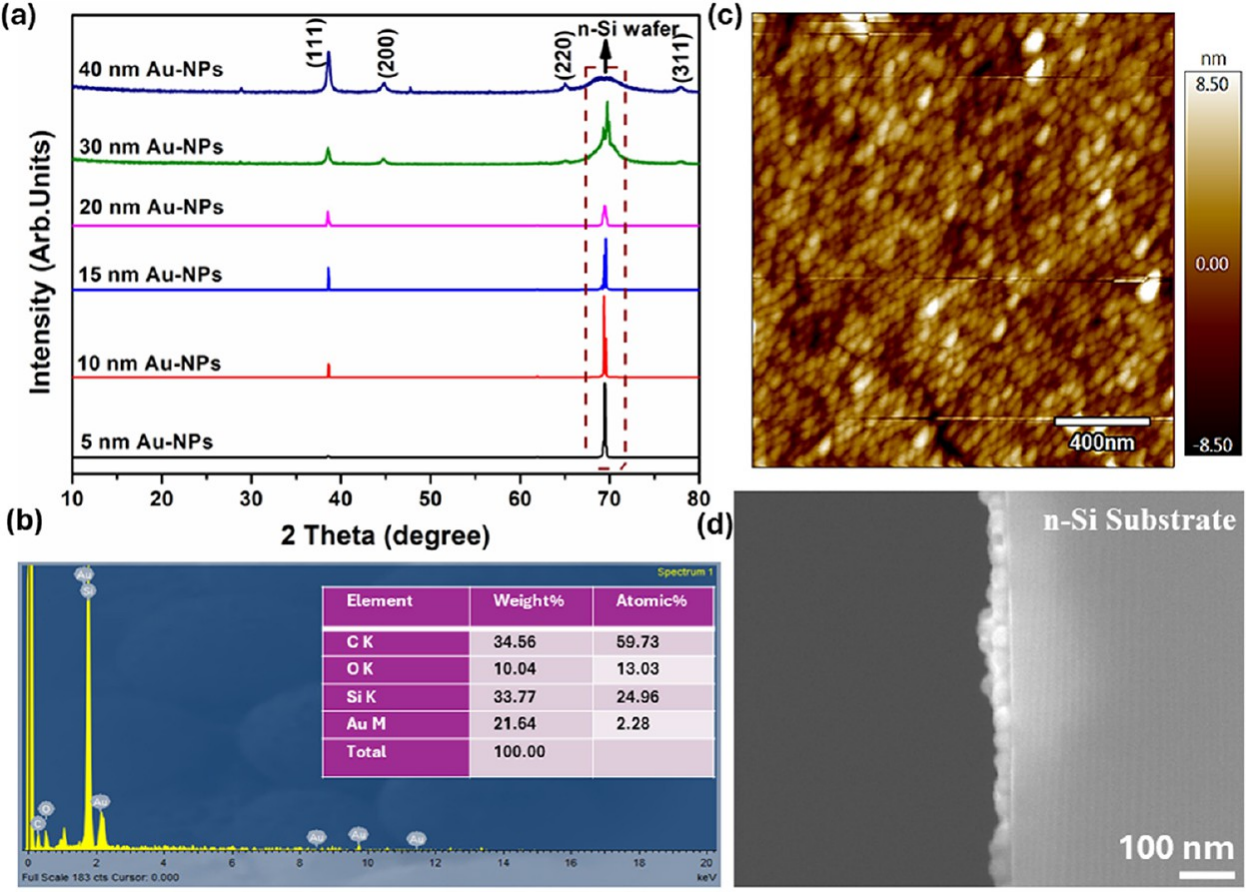
(a) XRD patterns of Au-NPs with different sizes (5 nm at 250 V, 10 nm at 250 V, 15 nm at 250 V, 20 nm at 500 V, 30 nm at 750 V, and 40 nm at 1000 kV) deposited on an n-type silicon (n-Si) substrate, (b) EDXS spectrum, (c) AFM morphology image for 20 nm sized Au-NPs and (d) SEM-cross section image for 30 nm sized Au-NPs on n-Si substrate.

In Figure 10a, the XRD patterns of Au-NPs of varying sizes (5 at 250 V, 10 at 250 V, 15 at 250 V, 20 at 500 V, 30 at 750 V, and 40 at 1000 kV) deposited on an n-type silicon (n-Si) substrate are presented. The observed differences in the intensity of the diffraction peaks provide insights into the distribution and deposition quality of the Au-NPs on the substrate. For the 5, 10, and 15 nm Au-NPs, the diffraction peak corresponding to the (111) plane is present but with low intensity. This indicates that while some Au-NPs are present and crystallized, their quantity or uniformity on the substrate is limited as compared to 20, 30, and 40 nm-sized Au-NPs. The high intensity of the diffraction peaks attributed to the n-Si substrate (around 69–70 degrees) suggests that the underlying silicon substrate is more prominently detected. This implies that the Au-NPs are not evenly distributed, leaving significant portions of the silicon surface exposed. In contrast, for the 20, 30, and 40 nm Au-NPs, the diffraction peaks corresponding to the (111), (200), (220), and (311) planes of Au-NPs are more intense and are well matched with standard JCPDS No. 00–04–0784. This indicates a higher density and better crystallinity of the gold nanoparticles. The lower intensity of the n-Si substrate peaks suggests that the surface coverage by Au-NPs is more uniform.

The extensive coverage of Au-NPs on the substrate reduces the level of exposure of the underlying silicon, leading to a decrease in the substrate peak intensity. The lower intensity of Au-NPs peaks and higher intensity of n-Si substrate peaks for 5, 10, and 15 nm Au-NPs imply that these smaller nanoparticles are not uniformly distributed on the silicon surface. This could be due to insufficient deposition, aggregation, or uneven spreading during the synthesis process. The higher intensity and presence of multiple diffraction peaks for 20, 30, and 40 nm Au-NPs suggest a more uniform and denser coverage of the gold nanoparticles on the substrate. The well-defined peaks indicate good crystallinity and a successful deposition process, which results in a more even and consistent nanoparticle layer. The well-distributed and highly crystalline 20, 30, and 40 nm Au-NPs are likely to exhibit superior performance in applications requiring high surface area, consistent electronic properties, and robust mechanical stability. This analysis underscores the importance of controlling the nanoparticle size and deposition methods to achieve optimal material performance. Also, the XRD analysis also shows the purity of the sample, as no extraneous peaks were observed, which could indicate the presence of impurities or secondary phases. Additionally, energy-dispersive X-ray spectroscopy (EDXS) analysis was performed to investigate the elemental composition of the samples. Figure 10b shows the EDXS spectrum, demonstrating the presence of Au, Si, C, and O in the prepared Au-NPs deposition on the n-Si substrate. The detected carbon is attributed to the carbon tape used during SEM analysis. The EDXS analysis confirms the absence of any additional or impurity phases, providing further support for the XRD results. This comprehensive analysis underscores the effectiveness of the synthesis and deposition process, highlighting the improved deposition quality with larger Au-NPs and the precise elemental composition of the samples.

The surface morphology properties of 20 nm-sized Au-NPs deposited on the n-Si substrate were examined using atomic force microscopy (AFM). The AFM image from Figure 10c provides detailed insights into the morphology of the nanoparticle deposition, illustrating the differences in the distribution and uniformity of the nanoparticles when subjected to a 500 V electric voltage during deposition. These nanoparticles form a well-arranged monolayer with very few voids and vacancies. The height scale bar shows minimal variation, highlighting the even distribution and better surface coverage achieved with the nanoparticles. This can be attributed to the effect of the electric field, which helps guide and settle the charged nanoparticles onto the substrate more effectively, reducing clustering and improving the overall uniformity of the deposition. Figure 10d presents the cross-sectional SEM image of 30 nm Au-NPs on the n-Si substrate, further confirming the formation of a monolayer, as supported by AFM analysis. These observations are crucial for optimizing the deposition process to achieve desired surface properties for various applications on sensors, coatings, and electronic devices.

**Figure 11:**
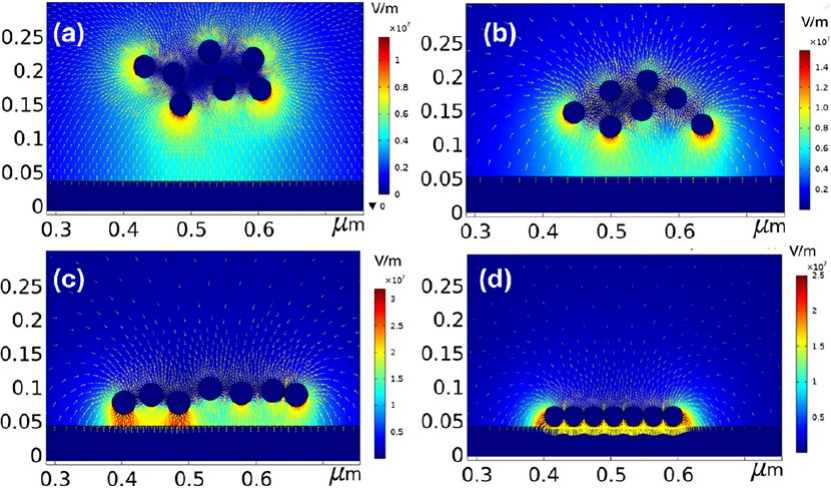
Finite element simulation of monolayer formation using COMSOL multiphysics software for 30 nm sized Au-NPs at 750 V deposition on the n-Si substrate. (a) Electric field distribution around randomly scattered charged Au-NPs. (b, c) Au-NPs converge due to the vertical electric field. (d) Au-NPs arranged into a monolayer on the silicon substrate.

To delve deeper into the mechanics of monolayer formation, we constructed a finite element simulation model using COMSOL Multiphysics software, as demonstrated in Figure 11a–d. The simulation aimed to capture the dynamic interactions between the electric field, the negatively charged Au-NPs, and their movement toward the substrate under an applied electric field. In Figure 11a, the electric field around the scattered 30 nm sized Au-NPs in the colloidal solution is depicted. Initially, the Au-NPs are randomly distributed, and the electric field is relatively uniform but intensifies near the nanoparticles due to their high surface charge. As shown in Figure 11b, c the externally applied voltage and the negative zeta potential of the Au-NPs lead to their gradual alignment. The electric field surrounding the Au-NPs is extremely strong, on the order of 1 × 10^7^ V/m, signifying a high surface charge density. This high surface charge density results in an extremely high electrostatic force per unit area (*F*) on the nanoparticles, *F* = σ*E*, where σ is the charge density on the Au-NPs, which drives their movement toward the silicon substrate. As the Au-NPs descend under the influence of the applied electric field, solvent evaporation plays a crucial role. The decreasing solvent volume increases the concentration of nanoparticles, enhancing their interaction with the electric field. Concurrently, the electric repulsion between neighboring Au-NPs increases, but this repulsive force is counterbalanced by the surface tension exerted by the citrate layer on the nanoparticles. This citrate layer acts as a stabilizing agent, preventing the nanoparticles from aggregating too quickly while maintaining sufficient spacing between them to form an orderly structure. When the Au-NPs reach the surface of the silicon substrate, as shown in Figure 11d, the interplay between the electric field and the surface tension allows the nanoparticles to form a well-ordered monolayer. The balance between the lateral repulsive forces and the vertical electric force is critical in achieving this uniform arrangement. Additionally, the gravitational force and Brownian motion also influence aggregation changes with NP size and electric field. Smaller nanoparticles, due to their lower mass, are more susceptible to Brownian motion, which can counteract the effects of gravity but is more easily overridden by a strong electric field. Larger nanoparticles experience stronger gravitational pull, which may aid in their sedimentation, complementing the effect of the electric field. However, their reduced Brownian motion and lower zeta potential compared to smaller NPs reduce the lateral repulsion, promoting faster aggregation and larger, more compact assemblies.

**Figure 12:**
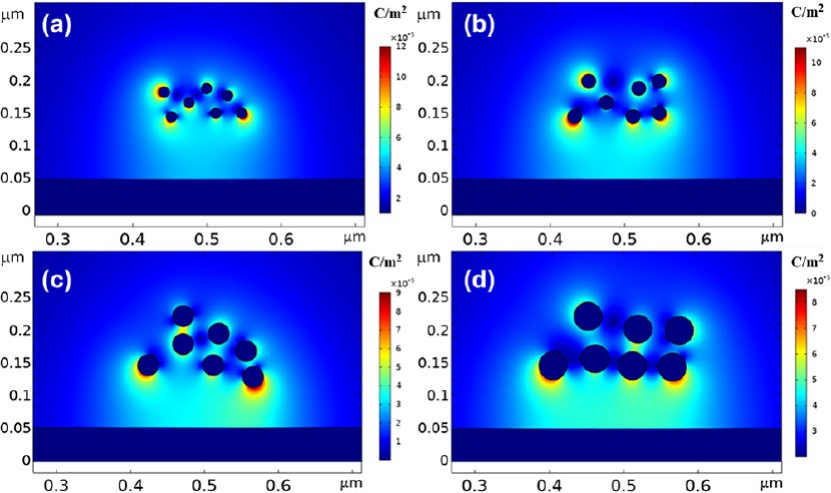
COMSOL multiphysics simulation of surface charge density on Au-NPs of different sizes: (a) 15, (b) 20, (c) 30, and (d) 40 nm.

COMSOL Multiphysics software is also utilized to determine the charge density on the surface of Au-NPs, as demonstrated in Figure 12a–d. The simulation reveals that the surface charge density is highly dependent on the size of the nanoparticles. For smaller NPs, such as those with sizes of 15 and 20 nm, the charge density is significantly higher compared to larger particles like 30 and 40 nm. This size-dependent variation in surface charge density is crucial because it directly influences how each nanoparticle interacts with the applied electric field during the deposition process. This simulation provides valuable insights into the underlying physics of monolayer formation, highlighting the delicate balance of forces that governs nanoparticle alignment and deposition. Understanding these mechanisms is essential for optimizing the process to achieve consistent and high-quality monolayer structures, which are crucial for various applications in electronics, sensing, and nanotechnology.

## 4 Conclusions

In conclusion, this research has provided valuable insights into the size-dependent assembly behavior of Au-NPs under an electric field, ranging from 5 to 40 nm. The multidimensional analysis has elucidated significant findings. XRD analysis revealed distinct variations in crystallinity and distribution among different nanoparticle sizes, with smaller Au-NPs exhibiting less uniformity and crystallinity compared to larger ones. SEM and AFM imaging further underscored these differences, depicting pronounced clustering and surface irregularities for smaller Au-NPs, contrasting with the smoother and more homogeneous monolayers formed by larger nanoparticles. COMSOL simulations supported these observations, demonstrating enhanced alignment and settling of larger Au-NPs under the influence of the electric field and facilitating structured monolayer formations. These findings are crucial for optimizing the deposition process and enhancing the functional properties of the nanostructured materials. By understanding how nanoparticle size influences assembly dynamics and the resulting monolayer properties, this study paves the way for designing advanced nanostructures tailored for applications in electronics, coatings, photonics, and catalysis. Future research could delve deeper into refining deposition techniques and exploring additional factors such as surface functionalization to further enhance the performance and versatility of Au-NP-based nanostructures in various technological applications.

## References

[ref1] Murthy S. K. Nanoparticles in modern medicine: state of the art and future challenges. Int. J. Nanomed. 2007, 2 (2), 129–141. 10.2147/IJN.S2.2.129. PMC2673971 17722542

[ref2] Pileni M.P. Nanocrystal self-assemblies: fabrication and collective properties. J. Phys. Chem. B 2001, 105, 3358–3371. 10.1021/jp0039520.

[ref3] Deader F. A.; Abbas Y.; Rezeq M.; Qurashi A.; Al-Qutayri M.; Chan V. Size-dependent formation of Au-NPs monolayer under high electric field. In 2024 IEEE 24th International Conference on Nanotechnology (NANO), Gijon, Spain, 2024; pp. 21–24.

[ref4] Abbas Y.; Deader F. A.; Qurashi A.; Al-Qutayri M.; Chan V.; Rezeq M. Gold Nanoparticles Monolayer Based Field-Effect Molecular Sensors. Adv. Electron. Mater. 2024, 2400363 10.1002/aelm.202400363.

[ref5] Rezeq M. Nanotips with a single atom end as ideal sources of electron and ion beams: Modeling of the nanotip shape. Microelectron. Eng. 2013, 102, 2–5. 10.1016/j.mee.2012.02.014.

[ref6] Black C.; Murray C.; Sandstrom R.; Sun S. Spin-dependent tunneling in self-assembled cobalt-nanocrystal superlattices. Science 2000, 290 (5494), 1131–1134. 10.1126/science.290.5494.1131. 11073445

[ref7] Abbas Y.; Rezeq M.; Nayfeh A.; Saadat I. Size dependence of charge retention in gold-nanoparticles sandwiched between thin layers of titanium oxide and silicon oxide. Appl. Phys. Lett. 2021, 119 (16), 162103 10.1063/5.0063515.

[ref8] Deader F. A.; Abbas Y.; Qurashi A.; Al-Qutayri M.; Chan V.; Rezeq M. Electric field-driven self-assembly of gold Nanoparticle monolayers on silicon substrates. Langmuir 2023, 39 (44), 15766–15772. 10.1021/acs.langmuir.3c02351. 37879624 PMC10634370

[ref9] Tizani l.; Abbas Y.; Yassin A. M.; Mohammad B.; Rezeq M. Single wall carbon nanotube based optical rectenna. RSC Adv. 2021, 11 (39), 24116–24124. 10.1039/D1RA04186J. 35479053 PMC9036672

[ref10] Singh S.; Kumar V.; Romero R.; Sharma K.; Singh J. Applications of nanoparticles in wastewater treatment. In Nanobiotechnology in Bioformulations; Springer, 2019; pp. 395–418.

[ref11] Shipway A. N.; Katz E.; Willner I. Nanoparticle arrays on surfaces for electronic, optical, and sensor applications. Chem. Phys. Chem. 2000, 1 (1), 18–52. 10.1002/1439-7641(20000804)1:1<18::AID-CPHC18>3.0.CO;2-L. 23696260

[ref12] Puntes V. F.; Gorostiza P.; Aruguete D. M.; Bastus N. G.; Alivisatos A. P. Collective behaviour in two-dimensional cobalt nanoparticle assemblies observed by magnetic force microscopy. Nat. Mater. 2004, 3 (4), 263–268. 10.1038/nmat1094. 15048109

[ref13] Sekoai P. T.; Ouma C. N. M.; Du Preez S. P.; Modisha P.; Engelbrecht N.; Bessarabov D. G.; Ghimire A. Application of nanoparticles in biofuels: an overview. Fuel 2019, 237, 380–397. 10.1016/j.fuel.2018.10.030.

[ref14] Mishra R.; Militky J.; Baheti V.; Huang J.; Kale B.; Venkataraman M.; Bele V.; Arumugam V.; Zhu G.; Wang Y. Theproduction, characterization and applications of nanoparticles in the textile industry. Text. Prog. 2014, 46 (2), 133–226. 10.1080/00405167.2014.964474.

[ref15] Langmuir I.; Schaefer V. J. Activities of urease and pepsin monolayers. J. Am. Chem. Soc. 1938, 60 (6), 1351–1360. 10.1021/ja01273a023.

[ref16] Schulz F.; Lokteva I.; Parak W. J.; Lehmkühler F. Recent notable approaches to study self-assembly of nanoparticles with X-ray scattering and electron microscopy. Part. Part. Syst. Charact. 2021, 38 (9), 2100087 10.1002/ppsc.202100087.

[ref17] Matricardi C.; Hanske C.; Garcia-Pomar J. L.; Langer J.; Mihi A.; Liz-Marzán L. M. Gold nanoparticle plasmonic superlattices as surface-enhanced Raman spectroscopy substrates. ACS Nano 2018, 12 (8), 8531–8539. 10.1021/acsnano.8b04073. 30106555

[ref18] Denkov N. D.; Velev O. D.; Kralchevsky P.; Ivanov I.; Yoshimura H.; Nagayama K. Two-dimensional crystallization. Nature 1993, 361 (6407), 26 10.1038/361026a0.8421493

[ref19] Swierczewski M.; Bürgi T. Langmuir and Langmuir-Blodgett Films of Gold and Silver Nanoparticles. Langmuir 2023, 39 (6), 2135–2151. 10.1021/acs.langmuir.2c02715. 36739536 PMC9933884

[ref20] Oliveira O. N.; Caseli L.; Ariga K. The past and the future of Langmuir and Langmuir-Blodgett films. Chem. Rev. 2022, 122 (6), 6459–6513. 10.1021/acs.chemrev.1c00754. 35113523

[ref21] Al-Johani H.; Abou-Hamad E.; Jedidi A.; Widdifield C. M.; Viger-Gravel J.; Sangaru S. S.; Gajan D.; Anjum D. H.; Ould-Chikh S.; Hedhili M. N.; et al. The structure and binding mode of citrate in the stabilization of gold nanoparticles. Nat. Chem. 2017, 9 (9), 890–895. 10.1038/nchem.2752. 28837175

[ref22] Sun S.; Murray C. B.; Weller D.; Folks L.; Moser A. Monodisperse FePt nanoparticles and ferromagnetic FePt nanocrystal superlattices. Science 2000, 287 (5460), 1989–1992. 10.1126/science.287.5460.1989. 10720318

[ref23] Bigioni T. P.; Lin X.-M.; Nguyen T. T.; Corwin E. I.; Witten T. A.; Jaeger H. M. Kinetically driven self assembly of highly ordered nanoparticle monolayers. Nat. Mater. 2006, 5 (4), 265–270. 10.1038/nmat1611. 16547519

[ref24] Wen T.; Majetich S. A. Ultra-large-area self-assembled monolayers of nanoparticles. ACS Nano 2011, 5 (11), 8868–8876. 10.1021/nn2037048. 22010827

[ref25] Park J.; An K.; Hwang Y.; Park J.-G.; Noh H.-J.; Kim J.-Y.; Park J.-H.; Hwang N.-M.; Hyeon T. Ultra-large-scale syntheses of monodisperse nanocrystals. Nat. Mater. 2004, 3 (12), 891–895. 10.1038/nmat1251. 15568032

[ref26] Martin M. N.; Basham J. I.; Chando P.; Eah S.-K. Charged gold nanoparticles in non-polar solvents: 10-min synthesis and 2D self-assembly. Langmuir 2010, 26 (10), 7410–7417. 10.1021/la100591h. 20392108

[ref27] Park J.-W.; Shumaker-Parry J. S. Structural study of citrate layers on gold nanoparticles: role of intermolecular interactions in stabilizing nanoparticles. J. Am. Chem. Soc. 2014, 136 (5), 1907–1921. 10.1021/ja4097384. 24422457

[ref28] Mazloomi-Rezvani M.; Salami-Kalajahi M.; RoghaniMamaqani H.; Pirayesh A. Effect of surface modification with various thiol compounds on colloidal stability of gold nanoparticles. Appl. Organomet. Chem. 2018, 32 (2), e4079 10.1002/aoc.4079.

[ref29] Wagener P.; Schwenke A.; Barcikowski S. How citrate ligands affect nanoparticle adsorption to microparticle supports. Langmuir 2012, 28 (14), 6132–6140. 10.1021/la204839m. 22417054

[ref30] Santhanam V.; Liu J.; Agarwal R.; Andres R. P. Self-assembly of uniform monolayer arrays of nanoparticles. Langmuir 2003, 19 (19), 7881–7887. 10.1021/la0341761. 39354707

[ref31] Floate S.; Hosseini M.; Arshadi M. R.; Ritson D.; Young K. L.; Nichols R. J. An in-situ infrared spectroscopic study of the adsorption of citrate on Au (111) electrodes. J. Electroanal. Chem. 2003, 542, 67–74. 10.1016/S0022-0728(02)01451-1.

[ref32] Lee Z.; Jeon K.-J.; Dato A.; Erni R.; Richardson T. J.; Frenklach M.; Radmilovic V. Direct imaging of soft- hard interfaces enabled by graphene. Nano Lett. 2009, 9 (9), 3365–3369. 10.1021/nl901664k. 19591495

[ref33] Rodríguez-González B.; Mulvaney P.; Liz-Marzán L. M. An electrochemical model for gold colloid formation via citrate reduction. Z. Phys. Chem. 2007, 221 (3), 415–426. 10.1524/zpch.2007.221.3.415.

[ref34] Yadavali S.; Sachan R.; Dyck O.; Kalyanaraman R. DC electric field induced phase array self-assembly of Au nanoparticles. Nanotechnology 2014, 25, 465301 10.1088/0957-4484/25/46/465301. 25355725

[ref35] Zhang H.; Cadusch J.; Kinnear C.; James T.; Roberts A.; Mulvaney P. Direct Assembly of Large Area Nanoparticle Arrays. ACS Nano 2018, 12 (8), 7529–7537. 10.1021/acsnano.8b02932. 30004661

